# Perception of the ambiguous motion quartet: A stimulus-observer interaction approach

**DOI:** 10.1167/jov.21.13.12

**Published:** 2021-12-29

**Authors:** Charlotte Boeykens, Johan Wagemans, Pieter Moors

**Affiliations:** 1 Laboratory of Experimental Psychology, Department of Brain and Cognition, KU Leuven, Leuven, Belgium; 2 Laboratory of Experimental Psychology, Department of Brain and Cognition, KU Leuven, Leuven, Belgium; 3 Laboratory of Experimental Psychology, Department of Brain and Cognition, KU Leuven, Leuven, Belgium

**Keywords:** motion quartet, apparent motion, individual differences, spatial location, spatiotemporal distance

## Abstract

Visual perception is the result of a highly complex process depending on both stimulus and observer characteristics and, importantly, their interactions. Generating robust theories and making precise predictions in light of this complexity can be challenging, and the interaction of stimulus- and observer-related effects is often neglected or understated. In the current study, we examined inter- and intra-individual differences and the effects of a wide range of three stimulus characteristics (i.e., spatial distance, temporal distance, and spatial location). Our results indicate that not all individuals show the same group average stimulus-driven effects on the perception of a motion quartet and that these effects are not always equal across the entire stimulus range. Moreover, we observed that there are clear individual differences in spontaneous perceptual dynamics and that these can be overridden by some but not all stimulus manipulations. We conclude that considering different stimulus manipulations, different observers, and their interactions can provide a more nuanced and informative view on the processes governing visual perception. This study examines the effect of spatial distance, spatiotemporal distance, spatial location, and individual differences on the perception of the ambiguous motion quartet.

## Introduction

### (Meta-)theoretical context

**Figure 1.**
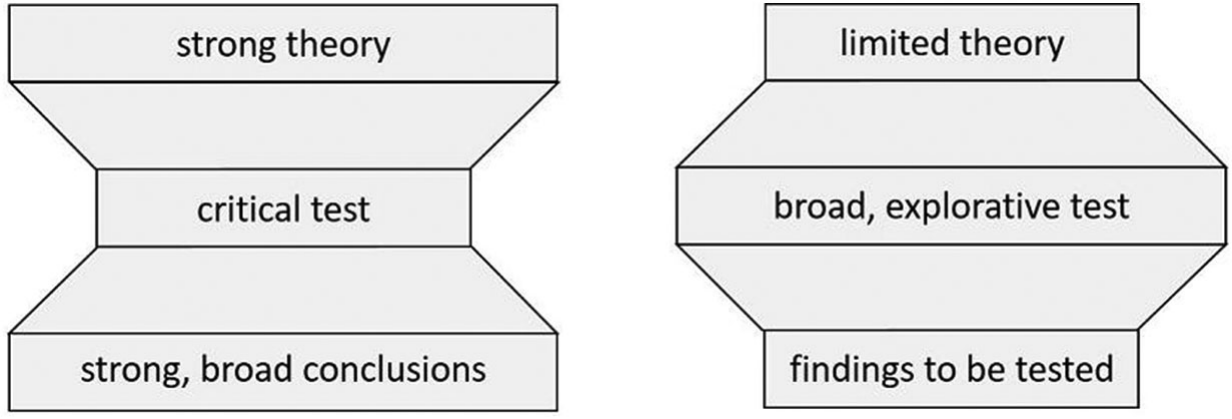
The hourglass versus reverse-hourglass approach. (Left) The standard hourglass approach, in which we start from a strong, broad theory, test precisely, and have strong, generalizable conclusions. (Right) The reverse-hourglass approach, in which we start from a limited theory, test broadly, and obtain exploratory findings, which should be followed up by further research for replication, extension, and refinement.

The normative approach for scientific experiments is to start from a strong theory in which a number of independent variables (e.g., stimulus size), predicted to influence a dependent variable (e.g., some measure of the percept), are specified in a conceptually coherent framework (Figure 1, hourglass). Subsequently, a critical design is chosen in order to test a hypothesis and advance the predefined theory by generating data that allow us to confirm or reject the hypothesis. This critical test typically focuses on a narrow range of manipulations of the independent variables. Accordingly, the hypothesis is tested as rigorously as possible, and conclusions can be generalized as broadly as the theory allows (Debrouwere & Rosseel, 2021; Meehl, 1990). This approach is optimal when you are dealing with a strong theory, meaning that the process studied is already well known and/or precise predictions can be made. However, there is considerable debate about the robustness of established knowledge of several cognitive functions (Davis-Stober & Regenwetter, 2019; Debrouwere & Rosseel, 2021; Fisher, Medaglia, & Jeronimus, 2018; Greenwald, Pratkanis, Leippe, & Baumgardner, 1986; Open Science Collaboration, 2015). Processes studied in behavioral science are influenced by multiple interacting factors, making it extremely difficult to generate robust theories and make precise predictions (Debrouwere & Rosseel, 2021; Open Science Collaboration, 2015).

Although vision science is known for relatively strong established knowledge and theory, many of the broader processes studied in vision science can be similarly challenging for generating robust theories and making precise predictions. Visual perception is the result of a highly complex process depending on both stimulus and observer characteristics and, importantly, their interactions. There are ample examples of studies in the literature reporting on the influence of stimulus or observer properties on perception. In addition to a wide variety of studies in which different stimuli are linked to different percepts in the average observer, research has shown that considerable individual variability exists when perceiving the same visual stimulus, as well. A good illustration of individual variability is provided by perceptual multistability, where the same visual stimulus elicits different percepts (usually two or three) when viewed multiple times or for an extended period of time (e.g., a few minutes). Accordingly, there is intra-individual variability in the perception of the same stimulus. Inter-individual differences in these perceptual dynamics have been well documented as well, and observers show reliable signatures in their “switching behavior” (e.g., the parameters of an observer's dominance duration distribution) (Brascamp, Becker, & Hambrick, 2018; Dieter, Sy, & Blake, 2017; Gallagher & Arnold, 2014).

Even well-known phenomena have been studied almost exclusively with an emphasis on either stimulus-related or observer-related effects in isolation. Indeed, keeping either the observer or the stimulus constant (or treating it as such) while manipulating the other can be a great tool to study perception. However, studying their interaction is crucial to further advance our understanding of visual perception. We may find that some interaction effects due to variability in stimuli and observers are not negligible. Yet, in order to focus on either stimulus or observer characteristics, the influence of observer or stimulus characteristics, respectively, and their interplay are often neglected or understated in research where actually both are (or should be) independent variables (Mollon, Bosten, Peterzell, & Webster, 2017). One may assume that the variability of the dependent measure (e.g., variability of percepts in different observers or variability in a stimulus set) is normally distributed, leading to the notion that the average describes the individual (stimulus or observer), although this is potentially not the case (Charest & Kriegeskorte, 2015; Davis-Stober & Regenwetter, 2019; Fisher et al., 2018; Wijnants, Cox, Hasselman, Bosman, & Van Orden, 2013). For example, stimulus-related effects of the average observer may not be informative for understanding any one observer in particular (Fisher et al., 2018). Alternatively, a sample may be taken from a very homogeneous group (e.g., all similar observers at one point in time or only one particular stimulus type), causing the variability in the sample to be normally distributed but compromising generalizability and making it potentially unrepresentative of the complexity of the processes that are studied. For example, findings about the perception of well-known stimuli may not be replicated when the stimulus is only slightly different (Wijnants et al., 2013). Similarly, as visual perception is a dynamic process, seemingly robust stimulus effects for one observer may change over time as well (Friston & Kiebel, 2009; Hamaker, 2012; Koenderink, 2019; Molenaar, 2004).

Perhaps some of our theories are not yet capturing the essence of the broader studied process, and perhaps we prevent further research progress when we keep measuring our dependent variables at particular isolated points in a non-uniform space that is influenced by multiple independent variables in ways that we are currently unable to predict. Think of trying to study a complex mechanism *M* in a group of individuals. We know that *M* has fluctuations due to internal factors and is influenced by external factors. Imagine that we measure the influence of a specific range of one external factor (e.g., 10–20 units) on *M* at a given point in time. At another point in time, we measure fluctuations in *M* due to internal factors. From these isolated studies, how should we predict *M* in the future? Some (curvi-)linear relationship between the external factor and *M* may exist within the scope of the studied units of the external factor (i.e., 10–20 units), but the effect of the external factor and its interactive effects may be very different outside this specific studied range. Even if we know what to expect on average within the studied scope of the external factor, internal factors or other external factors may still override the effect of the external factor at another point in time. Similarly, we may have an idea of the fluctuations of *M* due to internal factors, but surely this does not allow us to make precise predictions of *M* when the effect of an external factor is very strong. Moreover, internal factors and their interactive effects with external factors may depend on which different individuals we study and may differ considerably from one individual to another. Therefore, even though we considered multiple influencing factors in isolation, it might still be close to impossible to replicate findings or predict *M* precisely in the future. Alternatively, we could attempt to study the influence of a wider range of external factors, while simultaneously considering internal factors in multiple individuals. Accordingly, we can get a broader view of the complex mechanisms determining *M*. Importantly, we do not encourage drawing strong conclusions at the end of such an exploratory approach. These findings, because they are not the result of a critical test for a specific hypothesis, should be put to the test further on.

Likewise, when we study the effects of different stimulus and observer properties in isolation, and we only consider a narrow range of the stimulus and observer space, we may be missing valuable information. This may lead to theories lacking in explanatory or predictive value for particular observers and a wider range of stimuli. When our theories and knowledge of visual phenomena cannot account for this variability, difficulties in replication arise and the robustness of theories is compromised. In preparation for the current study, we considered multiple research questions concerning the perception of a multistable stimulus dependent on stimulus and observer properties, with corresponding potential operationalizations and designs according to the “hourglass” approach. However, we were confronted with its limitations in this context as mentioned above and concluded that it would be difficult to provide a critical test associated with a strong theory. Therefore, we found it worthwhile to consider a more exploratory approach, which may provide us with information on stimulus–observer effects that were not studied in combination before. This information may, in turn, shape our theories to better account for even main effects of simple stimuli. It implies that we will attempt to study a complex system as holistically as we can (instead of studying the effects of its components in isolation) in order to understand the complex system as a whole, as well as the effects of its components. Instead of adopting the standard hourglass approach (in which we start from a strong, broad theory, test precisely, and have strong, generalizable conclusions), we will start from a limited theory, test broadly, and have exploratory findings, which should be followed up by further research for replication, extension, and refinement (Figure 1, reverse hourglass).

### Empirical context

**Figure 2.**
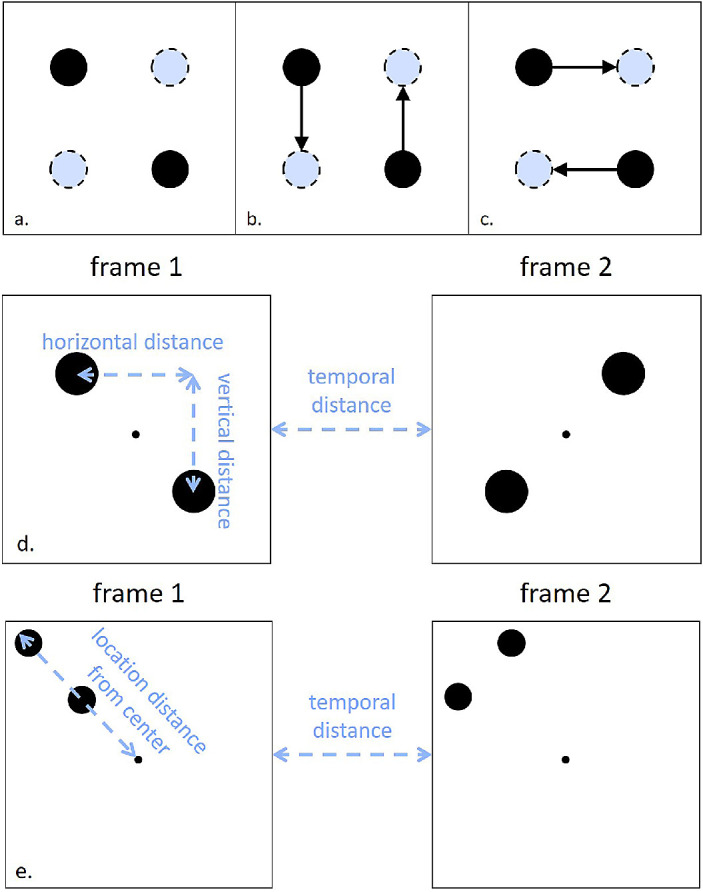
Ambiguous motion quartet stimuli. (a) The ambiguous MQ is depicted in the first frame in black and in the subsequent frame with dashed lines. In an alternating presentation of these two frames, apparent motion can be perceived. (b) Vertical motion percept of the ambiguous MQ. (c) Horizontal motion percept of the ambiguous MQ. (d) Ambiguous MQ in the Spatial Scale and Spatiotemporal Scale experiments. (e) Ambiguous MQ in the Spatial Location experiment.

For the current study, we considered observer- and stimulus-related effects on the perception of an ambiguous motion quartet stimulus (Figure 2). A motion quartet (MQ) is an ambiguous apparent motion stimulus that consists of two frames of two dots (Gengerelli, 1948). In the first frame, one dot is displayed in the upper left corner (or the upper right) relative to center and the other dot is displayed in the lower right (or the lower left) corner relative to center. In the second display, one dot is displayed in the upper right corner (or the upper left) and the other dot is displayed in the lower left (or the lower right) corner. When the two frames are presented successively, or with a sufficiently short time delay in between, observers can see apparent motion between the dots in the separate frames. The influence of several stimulus characteristics on the perception of the MQ has been studied for a long time, and we will briefly review some important findings. Although individual differences have almost always been reported, the focus remained on obtaining a general description of the perception of the MQ, assuming an absence of any noteworthy influence of observer characteristics. For example, the temporal distance between the two frames may elicit a difference in perception of the motion quartet (Ramachandran & Anstis, 1983). Previous studies have also documented a general vertical bias, indicating vertical apparent motion even when distances between dots across frames were equally large in both directions (Chaudhuri & Glaser, 1991; Genç, Bergmann, Singer, & Kohler, 2011; Gengerelli, 1948). Further studies have concluded that the vertical bias in the perception of the MQ may be due to the efficiency of motion processing within one hemifield and hemisphere compared with across hemifields and hemispheres (Chaudhuri & Glaser, 1991; Genç et al., 2011).

We were inspired by the studies of Mark Wexler and colleagues, in which perception of variations of different ambiguous motion stimuli were studied in different observers across multiple time scales (Wexler, 2018; Wexler, Duyck, & Mamassian, 2015). In the latter study, perception of the MQ was studied over time for stimulus presentations in different orientations. They found that observers exhibited particular patterns of percepts for these ambiguous stimuli. Interestingly, these patterns are robustly idiosyncratic and can evolve over time. A fitted model showed that the results were in accordance with the idea that perception is governed by continuously evolving internal brain states, which can be described as a random walk, due to external factors and internal neural dynamics. They concluded that, “Overall, these results exhibit the multidimensional complexity of internal states underlying the perception of even simple stimuli” (Wexler, 2018, p. 1).

The study of Wexler (2018) shed light onto the complex interplay of observer and stimulus characteristics in multistable perception, but its model provided only a description of the interaction of internal dynamics and the variation of one main stimulus characteristic. It is plausible that perceptual dynamics may be differentially affected by different external factors. If we strive for a robust, precise, and generalizable description and prediction of multistable perception, further exploration is necessary. In order to further determine how internal neural dynamics and external factors shape the perception of the MQ, we sought to explore the stimulus–observer space more broadly. This exploratory approach may provide us with more information on effects that were not studied in combination before, which may, in turn, shape our theories to better account for even main effects of simple stimuli. In the current study, starting from the findings in the classic literature about the perception of the MQ (Chaudhuri & Glaser, 1991; Gengerelli, 1948; Ramachandran & Anstis, 1983), we examined inter- and intra-individual differences and the effects of a wide range of three stimulus characteristics (i.e., spatial distance, temporal distance, and spatial location) as a case study in adopting the reverse-hourglass approach.

In addition, we introduce recurrence quantification analysis as a tool to quantify the characteristics of the temporal dynamics that different observers exhibit in response to different stimuli. A recurrence analysis looks into the number and duration of recurrences of a specific state (in our case, percept) in a dynamical system over time (Wallot, 2017). It is a nonlinear method of data analysis by means of which we aimed to explore the sequential effects of percepts in all observers.

## Spatial scale experiment

In the first experiment, we examined individual differences in the perception of the MQ across different horizontal and vertical spatial distances (Figure 2d). The previously reported vertical bias (Chaudhuri & Glaser, 1991; Genç et al., 2011; Gengerelli, 1948) has been demonstrated mainly by the prominence of vertical apparent motion in the average observer for a MQ with an equally large vertical and horizontal distance. Employing a similar MQ, but with a variable orientation, Wexler (2018) found more differences across observers, including a vertical or horizontal bias, as well as a clockwise or counterclockwise bias. Similarly, we examined the perception of a MQ with a variable spatial scale. By exploring perception of the MQ across different observers and a wider variety of spatial distances, we aimed to more extensively characterize conditions under which the previously reported vertical bias is present and thereby understand the mechanisms underlying the perception of the MQ more fully.

### Methods

#### Participants

Twenty-nine university students (one male; mean age, 18.9 years; *SD* = 2.23) participated in this study in return for course credit. All participants had normal or corrected-to-normal vision, provided informed consent, and were naïve with respect to the goals of the study. The study was approved by the Social and Societal Ethics Committee of KU Leuven (SMEC approval code G-2017 05 834).

#### Apparatus

All stimuli were displayed on a cathode-ray tube (CRT) monitor (GDM-F520; Sony, Tokyo, Japan) with a refresh rate of 60 Hz, a diagonal length of 50 cm, and a resolution of 1024 × 768 pixels. Participants were positioned in a dark room at a viewing distance of 57 cm from the monitor by means of a chin rest. Stimulus presentation and response registration were controlled by software programmed in Python using the PsychoPy library (Peirce, 2007).

#### Stimuli

All stimuli consisted of two white dots with a diameter of 0.78° (luminance, 76.8 cd/m^2^) and a central white fixation point with a diameter of 0.08° displayed on a gray background (luminance 17.9 cd/m^2^). One stimulus presentation consisted of two successive stimulus displays with a duration of 320 ms each (Figure 2d). There was no blank interval between these two displays; therefore, the stimulus onset asynchrony (SOA) within one stimulus presentation is identical to the stimulus display duration. In the first display, the dots were positioned in the top right and the bottom left relative to fixation. In the second display, the dots were positioned in the bottom right and top left relative to fixation (Figure 2). Horizontal and vertical distances between the centers of the two dots varied in discrete steps of 0.3° between 0.6° and 5.4°.

#### Procedure and design

The horizontal and vertical distances between dots displayed across the two displays varied on each trial and were selected from 289 (17 × 17) unique combinations of these distances in a randomized order. This resulted in 289 trials separated into three short runs. Observers were instructed to report the perceived direction of apparent motion after stimulus presentation on each trial. When they saw apparent motion between the top right and top left dot, and between the bottom left and bottom right dot, they indicated that they saw “horizontal”^1^ apparent motion. When they saw apparent motion between the top right and bottom right dot, and between the bottom left and top left dot, they indicated that they saw “vertical” apparent motion. During the inter-trial interval, the fixation point was displayed for 1000 ms.

#### Data analysis

All data analysis was conducted in R 3.6.0 (R Foundation for Statistical Computing, Vienna, Austria). Essentially, the data consist of a single measurement of an observer's percept for each unique stimulus (determined by the horizontal and vertical distance between the dots). Proportions of vertical versus horizontal perceptual reports were derived from these data, providing a coarse representation of perceptual bias. Our goal was to quantify an observer's tendency to perceive the stimuli more in either the horizontal or vertical direction, in relation to horizontal and vertical distance. In order to do this more precisely, we fitted a generalized linear model with a logistic link function to the data of the perceptual responses of each observer. Intuitively, this boils down to estimating a multidimensional psychometric function. We included linear and quadratic effects of both vertical and horizontal distance to assess how the perceptual responses changed in function of vertical and horizontal distance. Based on the estimated coefficients of this model, we derived a curve of subjective equality across all vertical and horizontal distances (i.e., the estimated transition curve between horizontal and vertical percepts, made up from the estimated point of subjective equality [PSE] along horizontal and vertical distances). Furthermore, the model coefficients were used to quantify the size of the transition zone between horizontal and vertical percepts (i.e., the inverse steepness of the psychometric function) and change/stability of the aspect ratio between horizontal and vertical distance at the PSE along the curve of subjective equality (i.e., the significance of quadratic effects).

### Results and discussion

**Figure 3.**
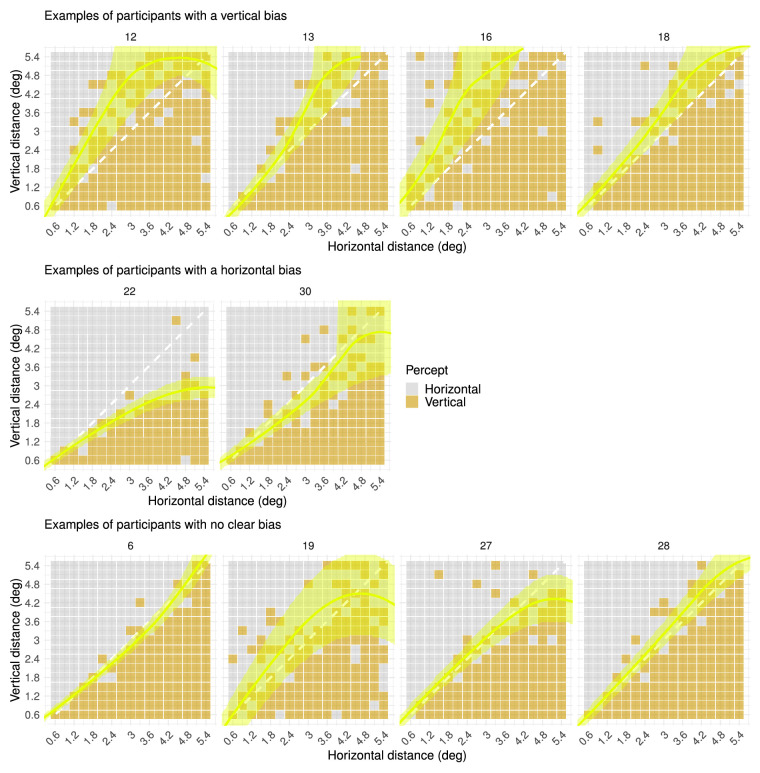
Percepts of ambiguous MQ across spatial scale (Spatial Scale experiment). Each plot represents all perceptual reports for one participant across all different vertical and horizontal distances. Each colored square in a plot represents one perceptual report, which may be horizontal or vertical. The yellow overlay represents the transition zone between horizontal and vertical percepts across spatial distances, derived from a fitted generalized linear model. From top to bottom, the figure shows examples of participants with a vertical bias, a horizontal bias, and no clear bias. The small number on top of each plot indicates the participant number. Plots for all participants can be found in Supplementary Figure SA (https://osf.io/tahqw/?view_only=f0b5de9a282140fa973df0763529b720).

**Figure 4.**
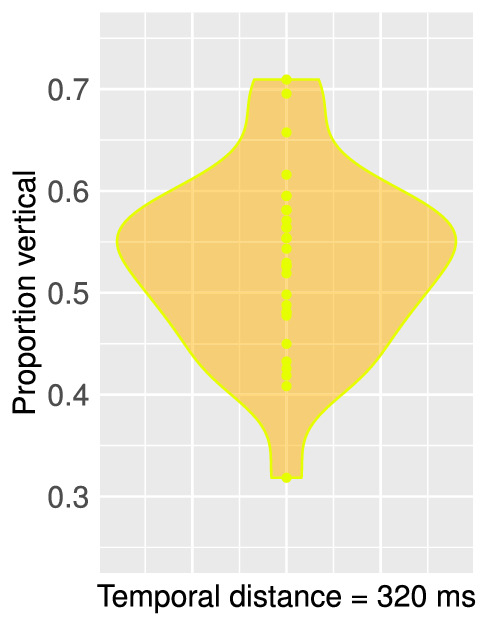
Density plot of proportions of vertical percepts per participant (Spatial Scale experiment). Each dot inside the density plot represents one participant. There are more participants with a vertical bias, as indicated by the relatively heavy top part of the density plot. However, there appear to be substantial individual differences, ranging from a vertical to a horizontal bias.

Visual inspection and proportions of perceptual reports over different horizontal and vertical distances show that there are substantial individual differences in perceptual biases of the ambiguous MQ (Figures 3 and 4; Supplementary Table S2 and Supplementary Figure SA for all participants). Perceptual bias here is defined as a tendency to perceive apparent motion between dots in one direction when the physical distance between these dots in this direction is not the shortest option available (i.e., not grouping by proximity). A large subset of the individuals showed a vertical bias, which was also reported in previous studies (Chaudhuri & Glaser, 1991; Gengerelli, 1948). However, unexpectedly, based on the existing literature, an almost equally large subset of individuals seemed to have no clear bias at all, approximating grouping by proximity. Moreover, a smaller number of individuals even showed a horizontal bias, which is in accordance with the findings for a different MQ in the study of Wexler (2018) but which had not been pointed out in other studies before. These results indicate that individual differences in perception of the MQ might be more important than previously assumed. In principle, this variability in perceptual bias extends far enough to yield subgroups differing categorically in perceptual bias (i.e., vertical, none, horizontal). However, individual variability in perception of the MQ might be best described on a continuous scale. Either way, further exploration of this variability will provide new insights that would be hidden when only considering a ubiquitous vertical bias. Moreover, we did not vary the order of both frames of the MQ stimulus, although there might be even more individual variability when this stimulus factor is also taken into account (Wexler, 2018). There are various possible explanations for the observed individual variability in perceptual bias, ranging from individual differences in low-level processing of visual input to differences in voluntary control of perception and perceptual reports. However, the influence of voluntary control is most probably limited, as Ramachandran and Anstis (1983) found that participants could not change their perception of a MQ by will for stimuli with an SOA shorter than 465 ms, and Wexler (2018) found that the effect of voluntary control on perception of a MQ was significant only for SOA longer than or equal to 400 ms.

**Figure 5.**
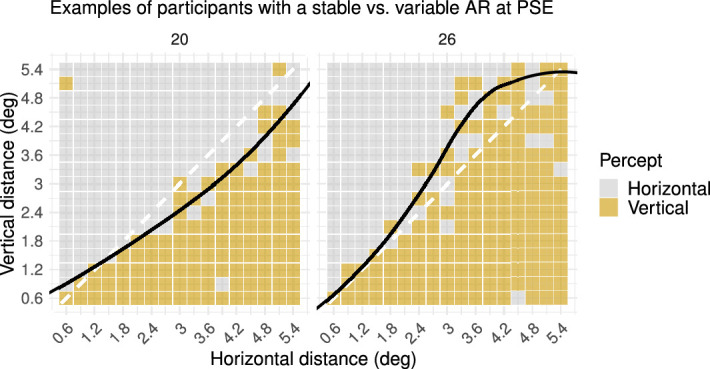
Percepts of two exemplary participants with a stable versus variable AR at PSE (Spatial Scale experiment). The black line in the plot represents the curve of subjective equality (i.e., PSE across different spatial distances), which was derived from the fitted generalized linear model. (Left) A black line that remains relatively linear across spatial distances indicates that the AR at PSE was stable across spatial distances. (Right) A black line that is relatively curvilinear across spatial distances indicates that the AR at PSE was variable across spatial distances.

As described in the Data analysis section, we fitted a generalized linear model and estimated transition curves from horizontal to vertical percepts across different vertical and horizontal distances. Visual inspection of these transition curves and the individual variability in (non-)significance of the quadratic effects in the model imply that there are individual differences in the stability of perceptual bias, or the aspect ratio (AR) at their PSE, over a spatial scale (Figure 5, Supplementary Table S1). Whereas some individuals remain more or less constant in the extent of their perceptual bias (i.e., rather linear transition curves and nonsignificant quadratic effects), others seem to increase or decrease in perceptual bias over horizontal or vertical distances (i.e., nonlinear transition curves and significant quadratic effects). This interaction with spatial scale has not been studied or emphasized before, yet our findings indicate that spatial scale might be an important factor to take into account when discussing the strength of the perceptual bias.

The coefficients for vertical and horizontal distance in the fitted model also provided a measure for the size of the transition zone between both percepts across spatial distances. More specifically, one may interpret the fitted transition from horizontal to vertical percepts across spatial distances as a three-dimensional psychometric function, where the change from one perceptual state to another occurs not only across one stimulus dimension (such as vertical distance) but also across two stimulus dimensions (i.e., horizontal and vertical distance). A higher term estimate and steeper slope in both dimensions of this fitted psychometric function correspond to a smaller transition zone, or less variability in percepts around the transition curve from horizontal to vertical percepts.

**Figure 6.**
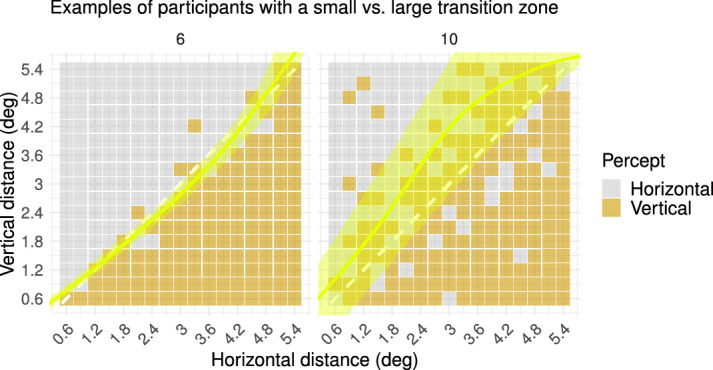
Percepts of two exemplary participants with a small (i.e., observer 6) versus large (i.e., observer 10) transition zone (Spatial Scale experiment). The yellow overlay in the plots represents the size of the transition zone (i.e., the range of spatial distances for which the participant has highly variable perceptual reports), which was derived from the fitted generalized linear model. (Left) A small transition zone indicates that the participant had an abrupt switch of perceptual reports across spatial distances, with little variability in perceptual reports on either side of the transition curve. (Right) A large transition zone indicates that the participant had a gradual switch of perceptual reports across spatial distances, with high variability in perceptual reports on either side of the transition curve.

**Figure 7.**
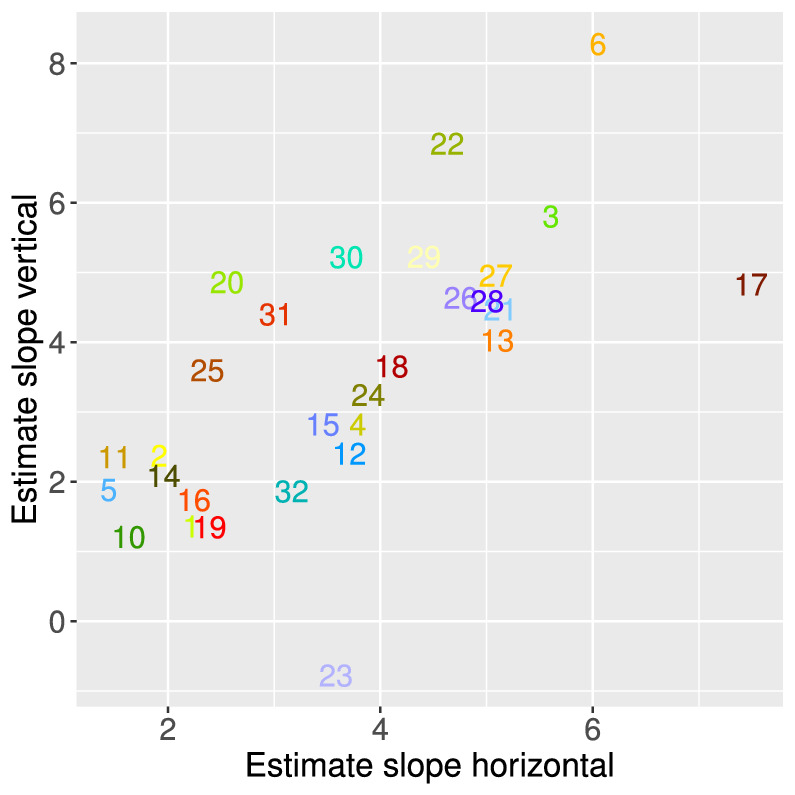
Estimates for the horizontal distance and vertical distance term in the generalized linear model for each participant (Spatial Scale experiment). Term estimates for each participant are indicated by the participant numbers in different colors. The higher the term estimate, the steeper the slope of the psychometric function and the smaller the transition zone. As can be seen in this plot, participants 6 and 10 (Figure 6) are on either ends of the range.

Here, we can also see considerable individual differences (see Figure 7). Figure 6 provides examples of two observers with a small (i.e., observer 6) vs. large (i.e., observer 10) transition zone, corresponding to two observers at either end of the scatterplot in Figure 7. The size of the transition zone may be interpreted in several ways, which are not mutually exclusive. One possibility is that, for a larger transition zone, there is a larger effect of internal noise, which can manifest itself in perception in several ways across the visual processing hierarchy (i.e., the stability or instability of the dynamic systems starting from processing visual input and leading to the organization of the percept, the decision process, and the generation of a response). In any case, the size of the transition zone has mostly been neglected in previous research, although individual differences in the size of the transition zone can be a highly valuable addition to what is known about the effect of perceptual bias on the perception of the MQ. It may be worthwhile to determine, for example, in which manner the perception of the MQ by an individual with a very small transition zone in combination with a strong vertical bias is driven by low-level and higher level factors. In some studies, a strong influence of perceptual bias has been interpreted as demonstrating a stronger top–down influence on perception compared with a more stimulus-driven influence of grouping by proximity on perception (Nikolaev, Gepshtein, & van Leeuwen, 2016). Yet, a strong perceptual bias might also be the result of a low-level distortion of perception (Carter & Cavanagh, 2007). Likewise, as mentioned before, the size of the transition zone may be the result of various factors along the visual–cortical hierarchy.

## Spatiotemporal scale experiment

In the second experiment, we examined individual differences in the perception of the MQ across different horizontal and vertical spatial distances, as well as different temporal distances (Figure 2d). Previous research has reported a differential perception of the MQ across different temporal distances, or SOAs (Ramachandran & Anstis, 1983). More specifically, Ramachandran and Anstis (1983) found that participants experienced hysteresis, with percepts of the MQ being less variable and less ambiguous, for stimuli with an SOA shorter than 400 ms. Again, it may be worthwhile to specify under which circumstances the impact of SOA is present and what it specifically entails, thereby contributing to a further understanding of the perception of the MQ.

### Methods

#### Participants

Twenty-two university students (four male; mean age, 18.95 years; *SD* = 1.84) participated in this study, which was otherwise identical to the Spatial Scale experiment with respect to participant-related information. Two participants were excluded because their random responses indicated that they did not report their percept.

#### Apparatus

The apparatus was identical to that of the Spatial Scale experiment.

#### Stimuli

The horizontal and vertical distances between the two dots in the stimuli varied in discrete steps of 0.4° between 0.6° and 5.4°. Stimuli were otherwise identical to those of the Spatial Scale experiment, except for an additional variability in temporal characteristics, with the duration of stimulus displays (i.e., SOA) ranging between 160 ms and 640 ms in steps of 160 ms. Both displays within one stimulus presentation had identical durations (Figure 2d).

#### Procedure and design

The temporal, horizontal, and vertical distances between dots displayed across the two displays varied on each trial and were selected from 676 unique combinations of these distances in a randomized order. This resulted in 676 trials, separated into seven short runs. The procedure was otherwise identical to that of the Spatial Scale experiment.

#### Data analysis

All data analysis was identical to that of the Spatial Scale experiment.

### Results and discussion

**Figure 8.**
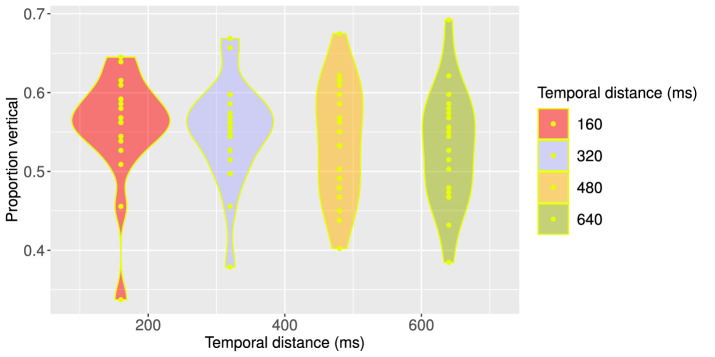
Density plot of proportions of vertical percepts per participant per temporal distance (Spatiotemporal Scale experiment). Each dot inside the density plot represents one participant. There were relatively fewer inter-individual differences and relatively more participants with a vertical bias for smaller temporal distances, as indicated by the relatively heavy top part of the first two density plots. However, there appear to be substantial individual differences for all temporal distances, ranging from a vertical to a horizontal bias. Percept plots for all participants can be found in Supplementary Figure SB, (https://osf.io/tahqw/?view_only=f0b-5de9a282140fa973df0763529b720).

Visual inspection of perceptual reports in all observers does not reveal clear differences in perceptual biases of the ambiguous MQ as a function of different temporal distances (Figure 8, Supplementary Figure SB for all participants). However, a one-way, repeated-measures ANOVA on the proportions of perceptual reports (Supplementary Table S4) indicates that the effect of temporal distance on perceptual bias is statistically significant, *F*(3,57) = 3.165, *p* = 0.031. More specifically, vertical perceptual bias was most prominent for shorter temporal distances (160 ms and 320 ms) and, on average, was less clear for longer temporal distances (480 ms and 640 ms). Moreover, there were more individual differences in perceptual bias for longer temporal distances, as can be seen in Figure 8. Nevertheless, for comparison, Figure 4 displays individual perceptual biases in the Spatial Scale experiment and suggests that for an SOA as short as 320 ms there can still be a significant amount of individual variability in perceptual biases. Our results show that temporal distance can be of crucial importance in the manifestation of perceptual bias in some observers and may have impacted results of various previous studies using an SOA of 100 to 250 ms (Chaudhuri & Glaser, 1991, Genç et al., 2011; Gengerelli, 1948).

Given the fact that there is less individual variability for shorter temporal distances, it is possible that perception of the MQ relying more on low-level processes is more homogeneous across individuals than when stimulus presentation time is sufficiently long to allow more elaborate mid- and higher level visual processing. However, shorter presentation times do not necessarily exclude higher level processes. As mentioned in the Spatial Scale experiment, there are various possible explanations for the observed individual variability in perceptual bias. Factors that may be at the basis of individual differences in perception of the MQ can be, for example, early processing of visual input, perceptual grouping, motion perception, or perceptual decision making, and further research is necessary to further disentangle these factors. Moreover, we cannot exclude the influence of voluntary control, or the possibility that participants willingly altered their percepts when the SOA was longer. However, the main source of variability is most probably not merely voluntary control, as we also found considerable variability in perceptual bias for stimuli with an SOA that is below the previously reported thresholds of 465 ms and 200 to 400 ms for voluntary control (Ramachandran & Anstis, 1983; Wexler, 2018).

**Figure 9.**
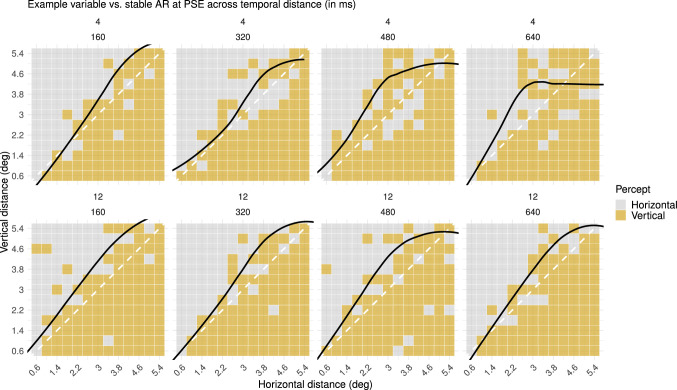
Percepts of two exemplary participants with no change versus change in the stability of the AR at PSE across temporal distances (Spatiotemporal Scale experiment). The black line in the plot represents the curve of subjective equality (i.e., PSE across different spatial distances) that was derived from the fitted generalized linear model. (Top) For this participant, the stability in the AR at PSE across spatial distances changed over temporal distances. (Bottom) For this participant, the stability in the AR at PSE across spatial distances remained relatively stable over temporal distances.

Individual differences in variability of the AR at PSE are more prominent for longer temporal distances. By means of the (non-)significance of quadratic effects in the fitted model we may derive that a subset of the tested individuals differed in the stability of perceptual bias over spatiotemporal distances (Figure 9, Supplementary Table S3). This rather complicated effect can be described as follows. There was little or no significant change in the AR at PSE across spatial distances for short temporal distances (160 ms) in any of the observers. However, for a subset of observers, change in the AR at their PSE across spatial distances was prominent for longer temporal distances (320, 480, and 640 ms). For the other observers, whether the change in the AR at PSE across spatial distances was small or large remained relatively stable across temporal distances. These results suggest that, aside from an increase in inter-individual variability for longer temporal distances, in some individuals, we saw an increase in intra-individual variability for longer temporal distances, as well.

**Figure 10.**
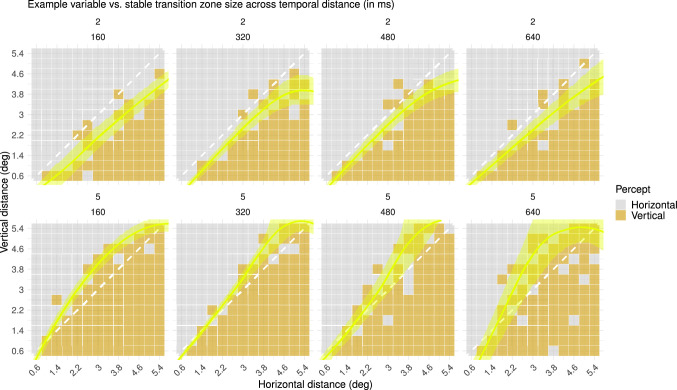
Percepts of two exemplary participants with no change versus change in the size of the transition zone across temporal distances (Spatiotemporal Scale experiment). The yellow overlay in the plots represents the size of the transition zone (i.e., the range of spatial distances for which the participant has highly variable perceptual reports), which was derived from the fitted generalized linear model. (Top) For this participant, the size of the transition zone remained relatively stable across temporal distances. (Bottom) For this participant, the size of the transition zone became larger across temporal distances.

**Figure 11.**
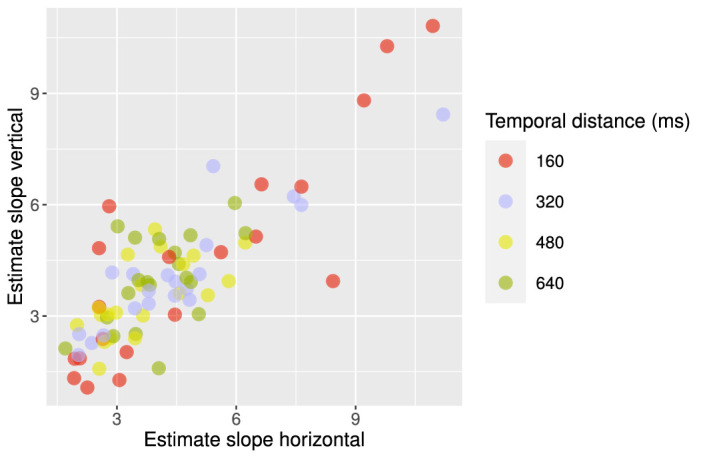
Estimates for the horizontal distance and vertical distance term in the generalized linear model for all participants per temporal distance (Spatiotemporal Scale experiment). Term estimates for each participant are indicated by dots in different colors per temporal distance. The higher the term estimate, the steeper the slope of the psychometric function and the smaller the transition zone. As can be seen in this plot, the smaller the temporal distance, the higher the term estimates.

Finally, individual differences in the size of the transition zone were more prominent for shorter temporal distances. A subset of the observers seems to have a small transition zone for a short temporal distance and larger transition zone for longer temporal distances, whereas other observers have a larger transition zone across all four temporal distances (Figure 10). We fitted the same generalized linear model and estimated transition curves from horizontal to vertical percepts across different vertical and horizontal spatial distances for all different temporal differences. Further inspection of individual differences in the term estimates revealed that this subset of observers indeed had a higher term estimate for shorter temporal distances and a lower term estimate for longer temporal distances (Figure 11), corresponding to a smaller and larger transition zone, respectively. The study of Ramachandran and Anstis (1983) reported that observers experienced less variability and ambiguity in their perception of a MQ with AR = 1 for shorter temporal distances. One way to compare this variability in percepts of the MQ in their study to our results is to look at the variability of percepts around the diagonal, as the AR is closest to 1 here. Because the transition zone largely overlapped with the diagonal in our results, the size of the transition zone could roughly reflect the ambiguity or variability of percepts around the diagonal. This comparison indicates that there was, indeed, an overall decrease in variability for shorter temporal distances, in accordance with the findings of Ramachandran and Anstis (1983). Interestingly, our results demonstrate that this overall decrease in variability, or overall decrease in the size of the transition zone, was driven only by a subset of the individuals with a smaller transition zone for shorter temporal distances (Figure 11).

Overall, these results suggest that not only were there more inter-individual differences in perceptual bias for longer temporal distances, but there were also more intra-individual differences in percepts along the transition curve for longer temporal distances. We may again speculate about which processes underlie an increase of the size of the transition zone for longer temporal distances, but further research should inquire into the specific roles of various stages of visual processing.

## Spatial location experiment

In the third and final experiment, we examined individual differences in the perception of the MQ across different stimulus locations (Figure 2e). Analogous to the study of Wexler (2018) and our first two experiments, the manipulation of spatial location induced some variability in the stimulus that was studied before, which allows us to characterize the conditions under which previously reported perception of the MQ is established. In combination with studying differences across observers, this will enable us to better understand the perception of the MQ. Moreover, previous research has stated that a vertical bias in perception of the MQ may be due to the efficiency of motion processing within one visual hemifield (Chaudhuri & Glaser, 1991; Genç et al., 2011). By means of varying the location of the MQ across the two hemifields, we can inquire into the robustness of this finding across stimuli and observers.

### Methods

#### Participants

Thirty-six university students (eight male; mean age, 18.8 years; *SD* = 1.27) participated in this study, which was otherwise identical to the previous experiments with respect to participant-related information.

#### Apparatus

The apparatus was identical to that of the Spatial Scale experiment.

#### Stimuli

The stimuli were slightly different from those used in the previous two experiments. They consisted of two successive displays of two white dots with a diameter of 0.5° (luminance 76.8 cd/m^2^) and a central fixation point with a diameter of 0.2° displayed on a gray background (luminance, 17.9 cd/m^2^). In the first display, the dots were positioned in the top right and the bottom left relative to the (not displayed) center between them. In the second display, the dots were positioned in the bottom right and top left relative to the (not displayed) center between them (Figure 2e). The diagonal distance between the centers of the dots was kept constant at 4.24°, resulting in a MQ with an AR equal to 1. Across trials the position of the two dots varied on an invisible 11 × 11 grid, spanning −6.75° to 6.75° in horizontal and vertical directions away from the fixation point, which was positioned in the center of the display. The duration of each display (i.e., SOA in our experiment) was 500 ms.

#### Procedure and design

The location of the MQ varied on each trial and was selected in a random order. This resulted in 121 trials executed in one run. Observers were instructed to report the perceived direction of apparent motion after stimulus presentation on each trial, identical to the first two experiments. They were instructed to keep their eyes on the fixation point, in order to perceive the MQ without any eye movements toward its location, which was further facilitated by employing randomly chosen and equally probable stimulus locations around the center of the display. An eye tracker was not employed in this experiment. During the inter-trial interval, the fixation point was displayed for 500 ms.

#### Data analysis

The data in this experiment also consist of a single measurement of an observer's percept for each unique stimulus (determined by the spatial location of the MQ). Proportions of vertical versus horizontal perceptual reports were derived from these data. Because there was no clear structure in the data based on spatial location (i.e., vertical and horizontal displacement of the MQ from center), no model dependent on spatial location was fitted to the data. In order to assess the effect of spatial location in a more coarse manner, we compared proportions of percepts across four visual fields, or quadrants (top-left, top-right, bottom-left, and bottom-right) in all participants by means of a test of equal proportions.^2^ Because the data consist of a single measurement per spatial location, measurement error would play a substantial role. In order to minimize the influence of measurement error and ensure that we did not miss any meaningful structure in the variability of perceptual reports, we also smoothed the data and repeated the test of equal proportions. The smoothing was obtained by means of sliding a 3 × 3 window across each perceptual report and replacing this value with the average of the nine perceptual reports in the window.

### Results and discussion

**Figure 12.**
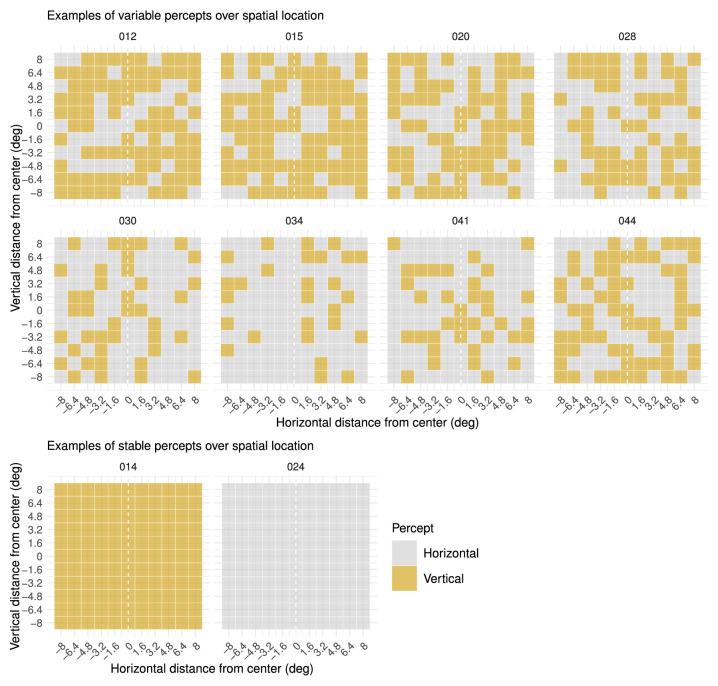
Percepts of ambiguous MQ across spatial location (Spatial Location experiment). Each different plot represents all perceptual reports for one participant across different vertical and horizontal distances. Each colored square in a plot represents one perceptual report, which may be horizontal or vertical. (Top) Examples of participants with variable percepts across spatial location (or time). (Bottom) examples of participants with a stable percept across spatial location (or time). Plots for all participants can be found in Supplementary Figure SC (https://osf.io/tahqw/?view_only=f0b5de9a282140fa973df0763529b720).

There were clear individual differences in (the dynamics of) perceptual reports of a motion quartet with AR = 1 presented across different locations in the visual field (Figure 12). Whereas most individuals were variable in their percepts over different spatial locations, a smaller subset of the observers appeared to be highly stable. Surprisingly, variability in perceptual reports did not seem to be driven by the location of presentation and seemed to occur irrespective of these stimulus manipulations. As can be seen by visual inspection of the examples in Figure 12 (see also Supplementary Figure SC for all participants and smoothed data), there seems to be no clear structure of percepts across spatial location. Results of the test of equal proportions also indicated that there was no significant difference in proportions of percepts compared across the four quadrants for any of the participants, except participant 040 (χ_3_ = 10.52, *p* = 0.015). However, the smoothed data did not yield a significant result for this participant or any other (Supplementary Figure SC). Therefore, we may conclude that there was no clear, meaningful influence of spatial location on the perception of this MQ with AR = 1. Again, we cannot exclude the influence of voluntary control, as the SOA was 500 ms. However, the absence of any dependence of percepts on spatial location was most probably not merely due to voluntary control, as the percepts in the spatiotemporal scale experiment were still clearly defined by stimulus characteristics when the SOA was as long as 640 ms. Interestingly, in contrast to the current findings, various previous studies have reported an effect of spatial location on the perception of visual stimuli (Finlayson, Neacsu, & Schwarzkopf, 2020; Moutsiana, De Haas, Papageorgiou, Van Dijk, Balraj, Greenwood, & Schwarzkopf, 2016; Schwarzkopf, 2019). This indicates that perception of different basic visual stimuli is differentially affected by location in the visual field, which would be worthwhile for further exploration. One of many possible distinctions here may be the difference between static and dynamic stimuli.

**Figure 13.**
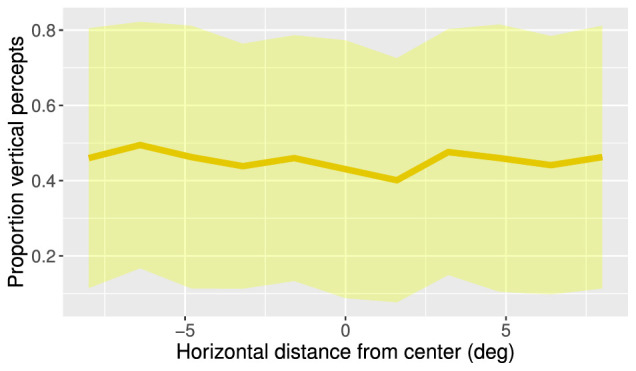
Average proportion of vertical percepts across horizontal distance from center (Spatial Location experiment). The thick line represents the average across participants, and the yellow overlay represents the range of 1 *SD* in both directions.

A remarkable finding here is that, on average, observers do not tend to report a vertical apparent motion percept more often when the motion quartet is presented in the vertical midline (i.e., horizontal distance of MQ from center is around zero) of the display (Figure 13). This contrasts with previous findings suggesting that the vertical bias was due to the hemifield(s) and hemisphere(s) of the presentation location (Chaudhuri & Glaser, 1991; Genç et al., 2011). Moreover, the data in this experiment indicate that there was no vertical bias at all (Figure 13). There may be several reasons for these conflicting results. In principle, it is possible that the observers in the current study differed in their perceptual bias from the observers in previous studies by chance. However, because the current study and that of Genç et al. (2011) both contain data from approximately 35 participants, this would be rather unlikely. Because we also observed a slight vertical bias on average in the first two experiments, it is more plausible that the specific stimulus properties in our study were substantially different from those of previous studies. For example, it is highly probable that the SOA of 500 ms had an impact on perceptual bias, as we found from the Spatiotemporal Scale experiment that longer SOA gave rise to more individual variability and, on average, a less prominent vertical bias. However, in contrast to the Spatial Location experiment, the stimuli with longer temporal distances (i.e., 480 and 640 ms) in the Spatiotemporal Scale experiment still yielded a vertical bias *on average*. This suggests that factors other than SOA are causing these conflicting findings. This is an important consideration, given that the MQ stimuli in the current study are not extensively different from the stimuli employed in previous research (other than SOA) and given that MQ stimuli employed in other studies often differ as much.

## Sequential effects of percepts

The results of the last experiment showed substantial individual differences in the variability of the perception of an identical motion quartet across space. However, perception did not seem to be influenced by stimulus location. As mentioned in the Introduction, perception is the result of both stimulus and observer characteristics and their interactions. So far, we have discussed the effects of stimulus manipulations (e.g., the overall effect of temporal distance on perceptual bias across all participants) and the observer-dependent interactive effects of these stimulus manipulations (e.g., the observer-dependent effect of temporal distance on the size of the transition zone). In the Spatial Location experiment, variability in perception was not driven by changes in a stimulus characteristic (i.e., spatial location) and we also did not see any observer-dependent interactive effects of this stimulus characteristic, so observer characteristics may be causing the variability.

Spontaneous dynamics in internal factors of the observer over time may be at the basis of the perceptual variability observed in the Spatial Location experiment. Moreover, if spontaneous dynamics are indeed a strong factor determining perception of the MQ, it is possible that they have played a role in the first two experiments assessing the influence of spatiotemporal characteristics, as well. In order to gain more insight into the temporal characteristics of the perceptual dynamics over the time course of the experiment, we employed recurrence quantification analysis. For comparison, we performed this analysis on all three experiments. A recurrence analysis looks into the number and duration of recurrences of a specific state in a dynamical system over time (Wallot, 2017). It has been employed in eye-movement research (Anderson, Bischof, Laidlaw, Risko, & Kingstone, 2013), where each state in the dynamical system of an observer's eye movements roughly corresponds to the same location of an eye movement. In the case of the current study, the dynamical system corresponds to an observer's multistable perception of the ambiguous stimulus, with the states corresponding to one of two possible percepts (vertical or horizontal apparent motion), recurrences of which correspond to an identical percept on particular trials. By means of the recurrence analysis, we aimed to explore sequential effects of percepts in all observers.

### Methods

**Figure 14.**
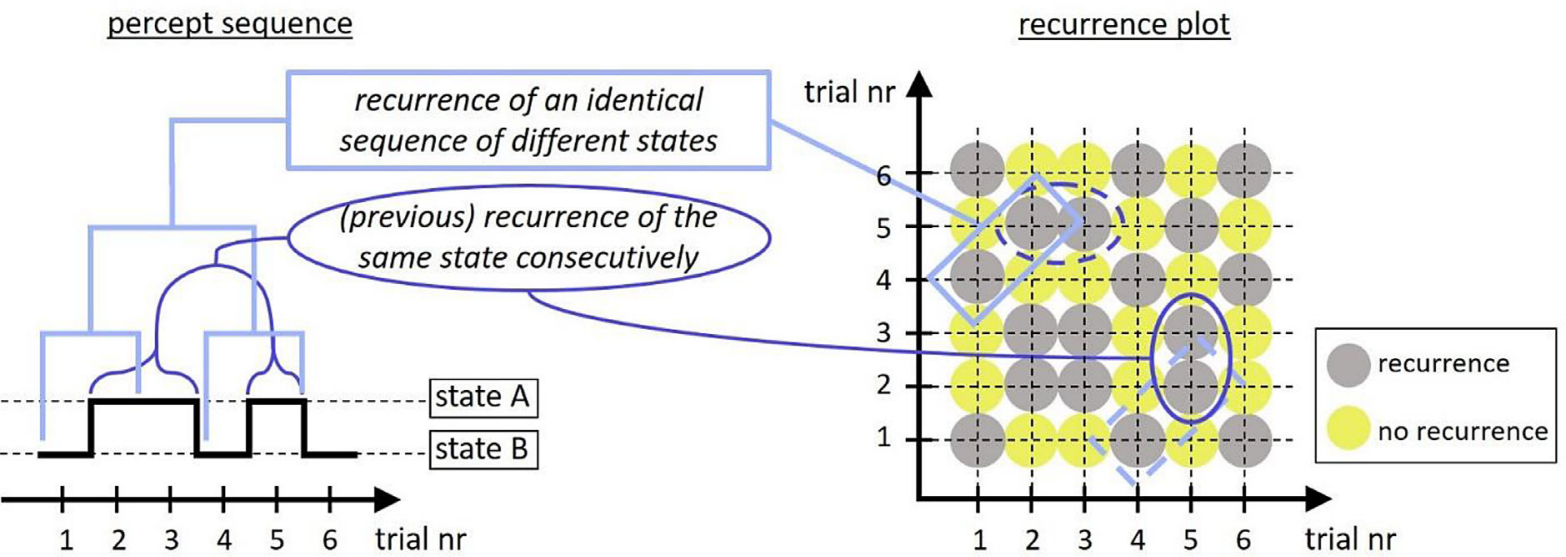
Example of a percept sequence and its corresponding recurrence plot. The light blue rectangles indicate the recurrence of an identical sequence of different states, as quantified by DET. The dark blue oval shapes indicate a (previous) recurrence of the same state consecutively, as quantified by LAM. A comparison of these two different types of sequential representations shows that a percept sequence is more suitable for displaying the specific states in a sequence, whereas the recurrence plot is more suitable for displaying the recurrence relationships among states in this sequence.

We examined individual perceptual dynamics by means of a recurrence quantification analysis (RQA) and its corresponding recurrence plots. A recurrence plot in the context of the current study is a plot with recurrence points displaying, for each moment within the measured time period, the times at which the dependent variable of interest has the same value as at that moment. For example, if we want to know whether the reported percept of an observer was identical at time point 1 and time point 5 (Figure 14, right), we can look for a recurrence point at the intersection on the plot of time point 1 on the *x*-axis and time point 5 on the *y*-axis, or vice versa (i.e., the plot is symmetrical along the diagonal).

A RQA evaluates visualizations in a recurrence plot and examines the number and duration of recurrences, which may be quantified by different measures. We will consider three of these measures, recurrence rate (RR), determinism (DET), and laminarity (LAM). RR is a measure of the density of recurrence points in the recurrence plot. DET is the proportion of all recurrence points on diagonals made up from multiple recurrence points. LAM is the proportion of all recurrence points on the vertical (or horizontal) lines made up from multiple recurrence points. Accordingly, the recurrence plot provides a direct visualization of (single) repetitions of the same state (i.e., recurrence points, which are measured by RR and correspond to the re-occurrence of a specific percept), multiple consecutive repetitions of the same state (i.e., vertical or horizontal lines of recurrence points, which are measured by LAM and correspond to the observer being “stuck” in one specific percept from one moment in time to the next), and repetitions of the same sequence of different states (i.e., diagonal lines of recurrence points, which are measured by DET and correspond to the re-occurrence of a pattern of percepts).

All of these visualizations and measures may also be visualized and measured in a more classic treatment of percept sequences, such as in Figure 14 (left). More specifically, a recurrence point (single re-occurrence of the same state and the derivative measure RR) may be seen as every instance of a certain state, excluding its first occurrence. The vertical or horizontal lines of recurrence points in a recurrence plot (i.e., multiple consecutive repetitions of the same state and the derivative measure LAM) may be seen as multiple consecutive instances of the same state. And finally, the diagonal lines of recurrence points in a recurrence plot (i.e., repetitions of the same sequence of different states and the derivative measure DET) may be seen as multiple occurrences of the same sequence of different states. However, a percept sequence is more suitable for displaying the specific states in a sequence, whereas the recurrence plot is more suitable for displaying the recurrence relationships among states in this sequence in a straightforward manner. Moreover, quantitative measures of sequential analysis can be obtained directly from the recurrence plots, whereas data rearrangement or supplementary analysis is necessary in order to obtain some of the equivalent measures from the classic percept sequence visualization.

### Results and discussion

**Figure 15.**
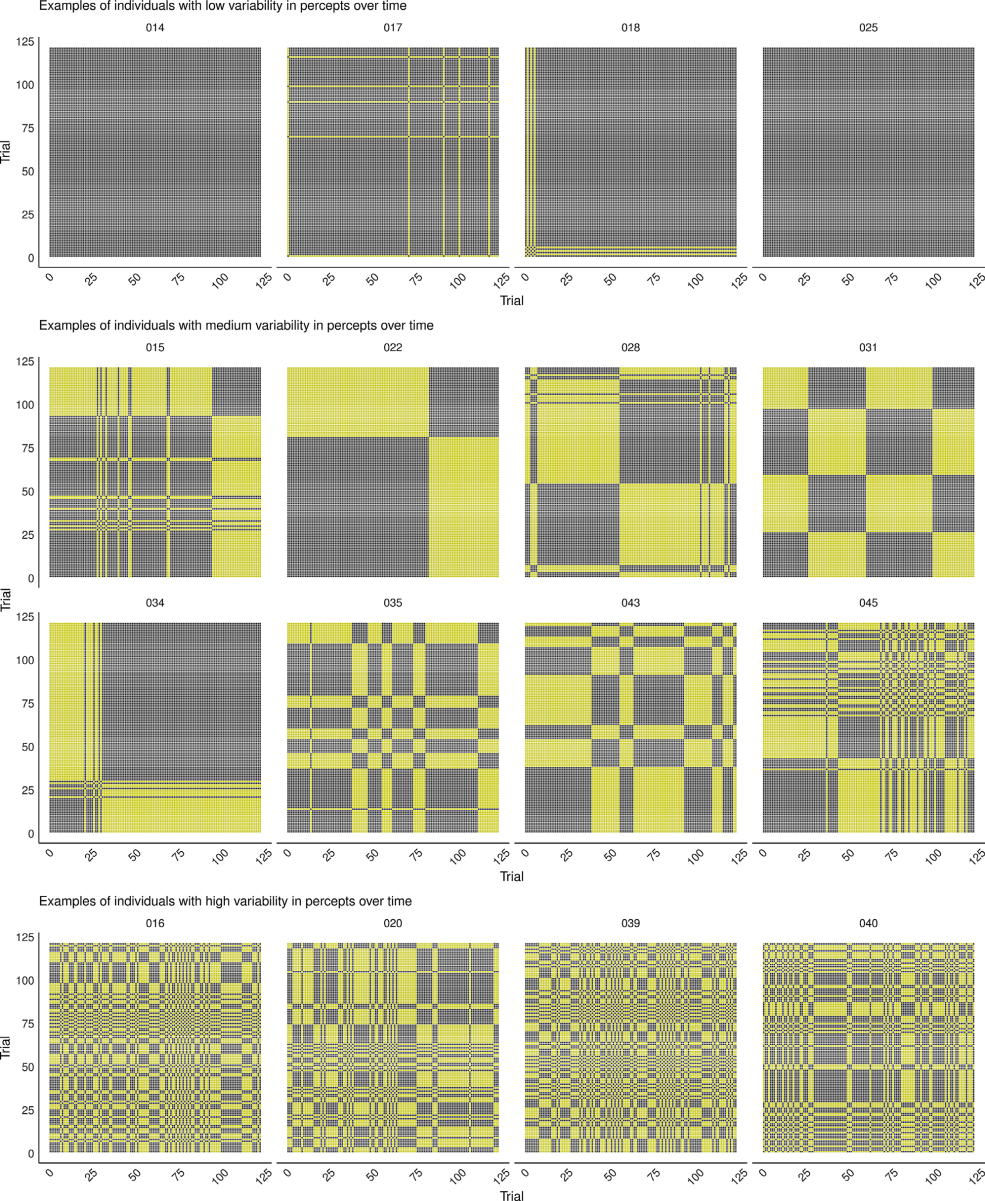
Examples of recurrence plots of the Spatial Location experiment. Each plot contains recurrence points displayed in gray, for each moment the times at which the perceptual report was the same as the one at that moment. For example, an identical reported percept of an observer at trial 1 and trial 50 is displayed by a gray recurrence point at their intersection on the plot. From top to bottom, the figure provides examples of individuals with a stable percept over time, individuals with a medium variability, and individuals with a high variability in percepts over time. Plots for all participants can be found in Supplementary Figure SE (https://osf.io/tahqw/?view_only=f0b5de9a282140fa973df0763529b720).

**Figure 16.**
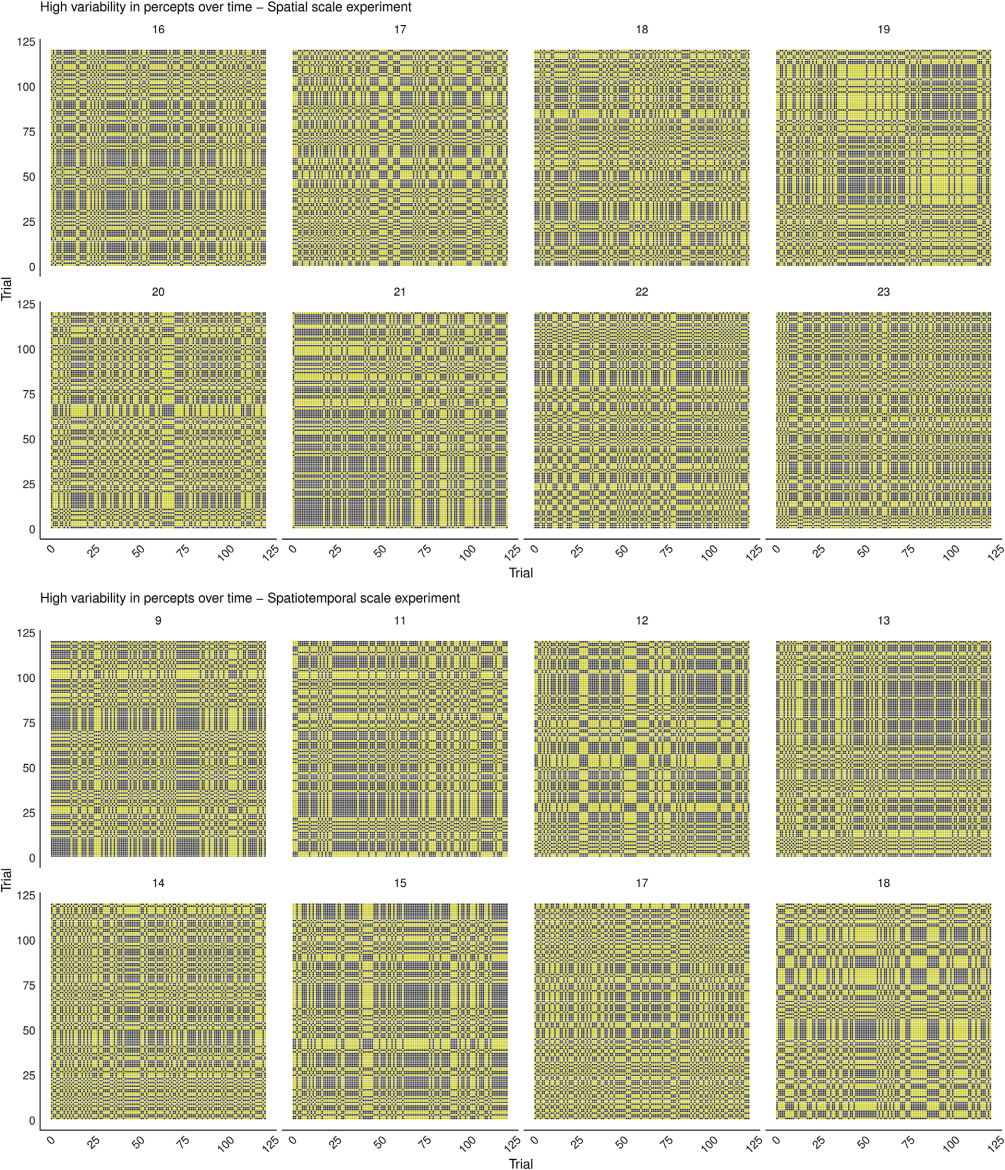
Examples of recurrence plots of the Spatial Scale and Spatiotemporal Scale experiments. Each plot contains recurrence points displayed in gray, for each moment the times at which the perceptual report was the same as the one at that moment. (Top) Examples of individuals from the Spatial Scale experiment, all with a high variability in percepts over time. (Bottom) Examples of individuals from the Spatiotemporal Scale experiment, all with a high variability in percepts over time. Plots for all participants can be found in Supplementary Figure SF (https://osf.io/tahqw/?view_only=f0b5de9a282140fa973df0763529b720).

The recurrence plots from the Spatial Location experiment (Figure 15, Supplementary Figure SE for all participants) clearly show individual differences in perceptual dynamics over time. Whereas some individuals were “stuck” in the same percept throughout the entire experiment, other participants showed slightly more variability in their percepts over time or had highly variable percepts over time. Even without looking at observer ID, we can derive that individuals that had a stable percept over spatial location (Figure 10, bottom) would have recurrence plots displaying a stable percept over time, as well (Figure 12, top), irrespective of which percept this was. However, without observer ID, it is not possible to determine which individual plots of percepts over spatial location (Figure 10) correspond to medium or high variability over time (Figure 12), as these plots do not provide information about the time (i.e., trial number) at which there was a stimulus presentation on each spatial location. Because the individual differences we saw in percepts of the MQ across the visual field did not seem to be determined by changes in stimulus characteristics (i.e., location), we may assume that these were due to individual differences in dynamics of internal factors. Interestingly, in contrast to the Spatial Location experiment, individual differences in perceptual dynamics over time seem to be completely absent in the Spatial Scale and Spatiotemporal Scale experiment (Figure 16, Supplementary Figure SF for all participants). All individuals in these experiments appear to be highly variable in their perceptual dynamics.

**Figure 17.**
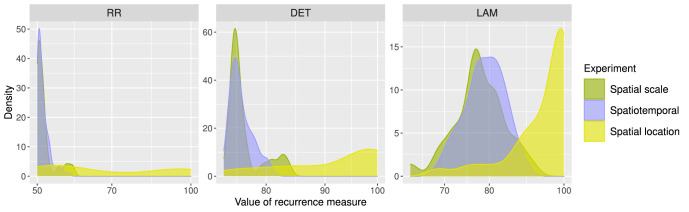
Density plots of RQA measures for all participants and all experiments. Each color in the density plots displays the values of the recurrence measure for all participants in the experiment corresponding to this color. Each density plot corresponds to a different RQA measure.

Likewise, in the results of the RQA we can see a similar difference between the Spatial and Spatiotemporal Scale experiments and the Spatial Location experiment. In Figure 17, density plots of observers with different values for different measures of the RQA are displayed. Whereas the observers had relatively evenly spread and highly variable RR values (i.e., measure of the density of recurrence points in the recurrence plot) in the Spatial Location experiment, they had more dense and homogeneously low RR values in the Spatial and Spatiotemporal Scale experiments. Similarly, whereas observers had more variable and higher DET values (i.e., proportion of all recurrence points on the diagonals, which correspond to the re-occurrence of a pattern) in the Spatial Location experiment, they had more homogeneously low DET values in the Spatial and Spatiotemporal Scale experiments. This implies that observers in the Spatial Location experiment were more likely to have the same percept multiple times (i.e., RR) and that they were more likely to have the same sequence of percepts multiple times (i.e., DET). Moreover, as the values of LAM (i.e., proportion of all recurrence points on the vertical lines, indicating identical values from one moment to the next) show, observers in the Spatial Location experiment were also more likely to have the same percept multiple times in a row, implying that observers tended to get “stuck” in one percept.

Yet, it is highly unlikely that all individuals in the Spatial Scale and Spatiotemporal Scale experiments were so homogeneous in the dynamics of their internal factors that these individual differences would be so small. Because spatial distance was altered on every trial and we saw a clear stimulus effect of spatial distance on perception in these experiments (i.e., percepts were clearly systematically driven by vertical and horizontal distances, as can be seen in Figure 3), a more probable explanation would be that individual differences in dynamics of internal factors were overridden by a stronger stimulus-driven effect of spatial and perhaps temporal distance. Likewise, Leopold, Wilke, Maier, & Logothetis (2002) found that individual differences in the perception of different ambiguous stimuli could be changed by manipulating the length of the inter-stimulus interval. Although the inter-individual differences in that study were left undiscussed, they can be observed in some of their figures. In the current study, the individual differences in dynamics of internal factors are most probably still present in the Spatial and Spatiotemporal Scale experiment, but they may have been cast to the background by a more dominant influence of stimulus manipulations. We cannot know for sure that temporal distances were a causal factor in the difference in recurrence plots for the Spatiotemporal Scale and Spatial Location experiment, as the recurrence plots from the Spatial Scale experiment did not seem to differ from those of the Spatiotemporal Scale experiment.

A consideration to be made is whether some individuals are more susceptible to stimulus differences overriding spontaneous internal dynamics than others. Accordingly, individual differences in the recurrence plots in the Spatial Location experiment may have been caused by individual differences in the influence of overriding stimulus effects, rather than individual differences in spontaneous internal dynamics. Following this line of thought implies that observers with the highest variability in percepts over time would also be the most susceptible to overriding stimulus effects. However, even when considering only the data of participants with high variability in percepts over time, the absence of any structure in percepts across spatial location remained. Therefore, it is indeed plausible that the individual differences observed in the Spatial Location experiment were driven by spontaneous internal dynamics.

Why is spatial scale a more disruptive stimulus influence for spontaneous internal dynamics than spatial location? One important difference between the Spatial Scale experiment and the Spatial Location experiment is the temporal distance between the two frames of the MQ, or SOA. However, even when we consider only the temporal distance of 480 ms (differing from the SOA of the Spatial Location experiment by only 20 ms) in the Spatiotemporal Scale experiment, we can see a clear structure of percepts dependent on spatial distance, whereas this is completely absent for the Spatial Location experiment. This indicates that the main stimulus properties of spatial scale versus spatial location were most likely at the basis of differences in the RQA. It would be very worthwhile for future studies to further investigate why spatio(-temporal) distances are more disruptive of spontaneous internal dynamics than spatial location.

We may draw a parallel between the RQA and other types of sequential analysis. Research on sequential effects, or history effects, has shown that there are many potential implications of the characteristics of previous stimuli and previous percepts on the perception of a stimulus in the current trial (e.g., priming, adaptation, hysteresis, perceptual stabilization) (Gepshtein & Kubovy, 2005; Murphy, Leopold, & Welchman, 2014; Pearson & Brascamp, 2008).

The effect of the previous percepts on the current percept is what we have looked into in the recurrence analysis. In the Spatial Scale and Spatiotemporal Scale experiments, we did not see any strong short-term or longer lasting effects of percepts in previous trials on the percept in the current trial. This can be derived from the patterns in the recurrence plots, as well as the RQA measures. More specifically, if there were a strong dependence on percepts in previous trials or an effect of perceptual stabilization, we would see a less variable pattern in the recurrence plots of these experiments and a less clear relationship of percepts to stimulus characteristics of the current trial, neither of which is the case. Moreover, as perceptual stabilization is usually seen for presentations of the same stimulus, the stimulus changes in each trial were probably also too large for any stabilization to occur.

However, in the Spatial Location experiment, there is a clear relationship between the previous percepts and the percept in the current trial for some observers. This can be derived from the low variability of the patterns in some of the recurrence plots, as well as the RQA measures. Perceptual stabilization might have happened, but only for the observers with a low variability in percepts across time. However, this would not explain why perceptual stabilization in these observers occurred regardless of stimulus location and did not occur in a location-contingent or retinotopic manner, as has been suggested in previous research on perceptual stabilization and memory (Chen & He, 2004; Knapen, Brascamp, Adams, & Graf, 2009; Murphy et al., 2014; Pearson & Brascamp, 2008; van Dam, 2010).

We may speculate about whether a strong relationship between previous percepts and the percept in the current trial is causal or whether there is a less direct relationship. For example, if there is a causal relationship between the previous percepts and the current one, a participant who has low variability over time will have a stronger effect of percept history than an observer who has high variability over time and therefore would be more likely to be “stuck” in one percept. On the other hand, if there is no causal relationship between previous percepts and the current one, a participant who has low variability over time may have an inherent temporary bias toward one percept, unrelated to percept history, and therefore would be more likely to be “stuck” in one percept. Importantly, either way, dynamic processes within the visual system of the observer, and not differences between external stimuli, appear to be at the basis of patterns of percepts, and individual differences in these patterns, in the Spatial Location experiment.

The recurrence analysis did not look into any effects of previous stimuli on the perception of the current stimulus. For the Spatial Scale and Spatiotemporal Scale experiments, the effect of the previous stimuli may have had an effect on the perception of the current MQ. However, no long-lasting effects (i.e., building up across multiple trials and lasting for multiple trials) of previous stimuli could have been present, as the order of stimuli was random and the eventual pattern of percepts was unmistakably formed by the current trial stimulus characteristics. Therefore, we should only consider the effects of the stimuli in the previous trial. Moreover, as percepts are almost exclusively influenced by stimulus characteristics of the current trial (e.g., a clear effect of horizontal distance), there can be a strong effect of the previous trial only around the diagonal (AR approximately = 1), as we see more variability in percepts and more individual differences there. A problem would arise if these differences around the diagonal, which partially constitute the individual differences of bias (horizontal, vertical, no bias), change in the AR around PSE (change, no change), and the size of the transition zone (small or large) would be due to merely a coincidental difference in the stimuli that were presented before the trials around this diagonal.

Because the differences between observers were made up from multiple trials and situated in a particular “region” of stimulus characteristics (e.g., a clustering of more horizontal percepts exactly around the diagonal is what constitutes an observer with a horizontal bias), it is quite unlikely that these differences were merely due to a coincidental difference in previous trials. However, as a proof of concept, we computed the median AR of the previous trials for all trials on the diagonal in the Spatial Scale experiment and found no diverging median AR for observers assigned to a particular perceptual bias, or for any observers in general. This indicates that the AR of the stimuli previous stimuli, or stimulus bias, was not a driving force for differences in perceptual bias. Therefore, we believe it is unlikely that differences between observers were due to differences between previous (random sequences of) stimuli in these experiments.

In the Spatial Location experiment, there was no clear effect of stimulus characteristics in the current trial at all, which makes any potential effects of stimulus characteristics of previous trials on the percept in the current trial even less likely. Therefore, there is no indication for previous stimuli being causally related to the differences between observers in this experiment. A coincidental difference of previous stimuli cannot be consistent enough across multiple (29, 22, and 36) participants to influence a difference between the Spatial Scale and Spatiotemporal Scale experiment vs. the Spatial Location experiment. Because there is also no reason to believe that differences in previous stimuli caused any differences between observers in any of the experiments, we do not believe that effects of previous stimuli were an important factor in any of the findings we discussed.

## Conclusions

In the current study, we aimed to illustrate the richness of information that can be uncovered when considering the interaction of various stimulus- and observer-related factors. We demonstrated that considering different stimulus manipulations, different observers, and their interactions can provide a more nuanced and informative view on the processes governing visual perception. First, we showed that not all individuals exhibited the same group average stimulus-driven effects on the perception of a MQ. Second, we showed that stimulus-driven effects are not always equal across the entire stimulus range. Third, we showed that there are clear individual differences in spontaneous perceptual dynamics and that these can be overridden by some (e.g., spatial scale) but not all (e.g., spatial location) stimulus manipulations. This study has provided a demonstration of how the interaction of stimulus and observer properties should indeed not be understated (Mollon et al., 2017). Moreover, we clearly observed that findings based on aggregations (i.e., perception across observers or stimuli) often fail to reflect the constituent processes in the individuals of which they are thought to be indicative (i.e., perception for a particular individual or perception of a similar but slightly different stimulus). As previously pointed out by Wexler (2018), visual perception of the MQ can be highly complex, depending on various stimulus and observer characteristics and their interactions. When this can be demonstrated for simple, well-known stimuli such as the MQ, they will most probably be at least as important for less extensively studied phenomena, as well. We were only able to shed more light onto this complexity thanks to the reverse-hourglass approach, and we would like to encourage more research to consider opting for a similar approach. The results of the current study may give rise to further, possibly more targeted, exploration, which will ultimately provide a foundation for a stronger, more comprehensive, and more robust theory.

## Footnotes

1 In the literature, perception of this apparent motion stimulus is often described as horizontal or vertical motion; however, two different kinds of percepts are possible under such a description. That is, some observers report seeing two dots moving independently horizontally or vertically, but others perceive the dots as belonging to a single object rotating clockwise or counterclockwise.

2 The test of equal proportions consists of a weighted sum of squared deviations between the observed proportions in each group and the overall proportion for all groups. The test statistic has an approximate χ^2^ distribution.
